# Ecological status of high altitude medicinal plants and their sustainability: Lingshi, Bhutan

**DOI:** 10.1186/s12898-016-0100-1

**Published:** 2016-10-11

**Authors:** Lakey, Kinley Dorji

**Affiliations:** 1 Horticulture Division, Department of Agriculture, Thimphu, Bhutan; 2 Renewable Natural Resources Research and Development Center, Bajo, Wangdue, Bhutan

**Keywords:** Wild harvesting, Medicinal plants, High altitude, Lingshi, Bhutan

## Abstract

**Background:**

Human beings use plants for a multitude of purposes of which a prominent one across the globe is for their medicinal values. Medicinal plants serve as one of the major sources of income for high altitude inhabitants in the Himalaya, particularly in countries like Nepal, and Bhutan. People here harvest huge volumes of medicinal plants indiscriminately, risking their sustainability. This paper attempts to identify some of the priority medicinal plant species harvested in the wild and assess their ecological status for their judicious utilization, and to help provide policy guidance for possible domestication and support strategic conservation frameworks.

**Results:**

Out of the 16 priority species identified by the expert group, collectors’ perception on ecological status of the priority species differed from survey findings. *Chrysosplenium nudicaule* (clumps) ranked as most threatened species followed by *Corydalis dubia*, and *Meconopsis simplicifolia*. *Onosma hookeri, Corydalis crispa and Delphinium glaciale* were some of the species ranked as threatened species followed by *Halenia elliptica* (not in priority list). Percent relative abundance showed irregular pattern of species distribution. High species evenness was recorded among *Nardostachys grandiflora*, *Chrysosplenium nudicaule*, *Saussurea gossypiphora* and Aconitum orochryseum with average species density of 8 plant m^−2^. *Rhodiola crenulata*, and *Dactylorhiza hatagirea* followed by *Meconopsis horridula* and *Meconopsis simplicifolia* were ranked as most threatened species with average species density of 0.4, 0.4, 5.6 and 6.0 plant m^−2^, respectively. The most abundant (common) species was *Onosma hookeri* (plant m^−2^). Species composition and density also differed with vegetation, altitude, slope and its aspects.

**Conclusion:**

Priority species identified by expert group were found vulnerable and patchy in distribution. Survey results and collectors’ perceptions tally to an extent. Some of the species (*Dactylorhiza hatagirea, Rhodiola crenulata, Meconopsis simplicifolia and Meconopsis horridula*) were critically low in plant density with less than a plant per m^2^ while *Delphinium glaciale, Fritillaria delavayi and Aconitum orochryseum* were confined to narrow altitude range. Collectors were aware that most species identified in priority list are threatened and existing harvesting plan are hardly implemented as it is not pragmatic. Moreover, major chunk of medicinal plants harvested remain unaccounted as illegal harvest and marketing seemed to occur across the borders. Policing and monitoring would continue to be a challenge given the rugged terrain and harsh climate. In-depth study and further monitoring of low density species is suggested to ensure its sustainability through long term strategy development.

**Electronic supplementary material:**

The online version of this article (doi:10.1186/s12898-016-0100-1) contains supplementary material, which is available to authorized users.

## Background

Over 422,000 plant species worldwide possess medicinal value [1] of which 52,885 species are traded globally. [2]. Wild resources serve as a main source (80–90 %) of the medicinal plant species. The Indian *Ayurvedic* system alone uses around 1250–1400 medicinal plants species of which almost 80 % are wild collected [3]. An ever-growing global botanical market compounds the pressure on plant resources. Larson and Olsen [4] reported that botanical plant market is worth US$ 20–40 billion and is increasing at an annual rate of at 10–20 %. Also, a report prepared by the Queensland Regional Forest Assessments Steering Committee [5] indicated that Australia alone exported $30 million worth native flora. It also reports an increase in the value of “bush-picked” materials that grew from $0.70 million to $2.8 million between the period spanning 1989–1993, and by 1998 the value of bush-harvested foliage from South-East Queensland alone has reached around $3 million.

A large proportion of Himalayan flora possesses medicinal value and the region is known as global centre for medicinal plants [3]. Around 5603 higher plant species are reported in Bhutan of which 600 species are known for medicinal properties [4]. Diverse ecological growing conditions and also altitude ranging from 100 to 7500 m above sea level (masl) favours the growth of diverse kinds of medicinal plants [3]. These medicinal plants not only play an important role by directly contributing to healthcare system but also serve as primary source of income [6] thus contributing to Gross National Happiness (GNH) [7]. Due to its richness in medicinal plants, Bhutan was known as Menjong (land of medicine/medicinal plant) [8]. In fact, medicinal plants used in Bhutanese traditional medicine (BTM) are known by different names based on altitude as “sngon sman” (higher elevation medicinal plants) and “Khrog sman” (lower elevation medicinal plants) [9]. It is due to availability of these diverse kinds of medicinal plants that the practice of indigenous medicine is rooted in Bhutanese culture and tradition. At present, Bhutanese traditional medicine has equal status to modern medicine with its unit spread all over the country [10, 11]. It is probably due to this spread of traditional medicine (TM) units within the country, awareness and treatment satisfaction among the Bhutanese people that raise demand for the TM services and collection pressure on native plants—compromising the sustainability of these medicinal plants.

Out of the several other factors that contribute to the decline of medicinal plants in Bhutan, unscientific harvesting poses increasing threat to their sustainability. Also, increased international market for medicinal plants lead to illegal harvesting, thereby reducing plant population in their natural habitat [12, 13]. However, much of the decline in other Trans-Himalayan region is attributed to loss of natural habitat [3]. Additionally, in the fragile ecosystems of the Himalayas these medicinal plants have become more vulnerable [13–16] to indiscriminate and unscientific harvesting, thereby demanding the need for sustainable management.

In Bhutan, about 300 species of medicinal plants are used in production of traditional medicine [17]. Annually, the Institute for Traditional Medicine Services (ITMS) uses over 18 tonnes of medicinal plants in their formulary, 85 % of which comprise species collected directly from the wild [17]. Precise and recorded figures on informal and illegal collection of medicinal plants for other purposes by local inhabitants are not available. With improved accessibility through motor roads and the growing demand for traditional medicine as well as rising need for medicinal plant resources in the wild from pharmaceutical agencies, collectors have now resorted to indiscriminate harvest that put enormous pressure on to the sustainability of this medicinal plants although most of the collecting areas in Bhutan still fall under protected areas (parks and sanctuaries).

Information on uses and conservation status of the medicinal plants in nearby Himalayan countries of South Asia are available [3, 18, 19] but there is very limited study conducted for medicinal plants in Bhutan. The imposing ruggedness of the Himalayas and the very limited human resources with associated agencies have not only made policing and enforcement of management and conservation regulations more difficult but also pose serious challenges in conducting scientific studies. Lack of scientific information on population dynamics or ecological demographics limits clearer understanding of the sustainability status of medicinal plants in Bhutan. Such information (distribution ecology, genetic diversity and their variation over space and time) form the basis of sustainable harvesting strategies and are integral to development of sound and adaptive management programs for prioritized medicinal plant species in the wild [2, 3, 20]. The aim of his study was to assess the ecological status of 16 priority (vulnerable) medicinal plant species in Lingshi by assessing collectors’ perceptions and population density survey.

## Methods

### Study area

**Fig. 1:**
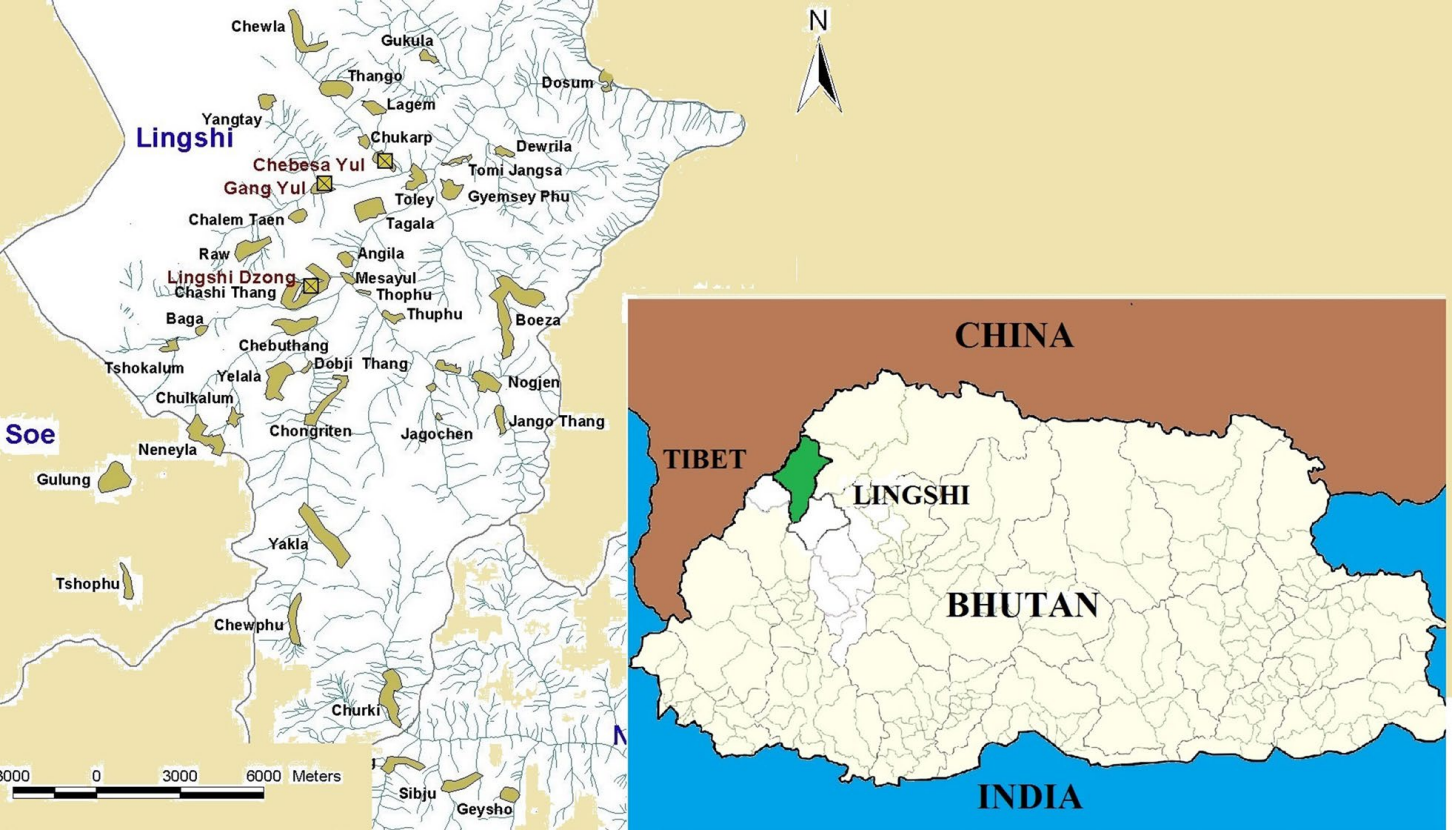
Map of Bhutan showing Lingshi highlighted (*green colour*) and sampling sites (*golden colour*)

Lingshi Dungkhag^1^ is located between the latitudes 27°35′13″ N to 27°54′40″ N and longitudes 89°14′ 51″ E to 89°38′44″ E within the Jigme Dorji Wangchuck National Park in north-west Bhutan, close to the border with Tibet (Fig. 1). Its administrative centre, Lingshi Dzong, lies approximately at 3500 m above sea level and a little over 3 days walk from the capital city, Thimphu. Lingshi has served as the main source of high-altitude medicinal plants for the ITMS in Bhutan for over 20 years. There are 11 small settlements in the region, the largest of which are Gangyul and Chebesa. The altitude ranges from 3406 to 5090 masl. The area remains covered with snow in winter months (January to March) and temperatures range from 0° (in winter) to 12 °C (in summer). The local economy revolves primarily around yak herding, with most community members spending the winter in their villages and summer in high-altitude grazing pastures, living in temporary camps.

### Vegetation cover

Vegetation in the region comprises a mixture of alpine meadows (often with scattered *Juniper* spp. at lower altitudes), *Rhododendron* scrub (including substantial areas of *Rhododendron anthopogon* and *Rhododendron setosum*), and low woodland dominated by *Betula*, *Salix* and *Rhododendron* spp. Grassland generally dominates on south-facing slopes and in the valleys, whilst scrub and woodland communities are commonly prevalent on north-facing slopes.

### Soil features

Soil physical characters in the study sites vary with aspect, altitude, slope and vegetation cover. Most area under survey falls in high altitude meadow soil category with high humus content and black in colour. The soil cover gets shallow as one move higher up and usually course textured mixed with pebbles and prone to erosion.

### Experimental design and data collection

The study followed “Guidelines on the conservation of medicinal plants” in identification of experts published by World Health Organization (WHO), International Union for Conservation of Nature and Natural Resources (IUCN) and World Wildlife Fund (WWF) [24]. The Council for Renewable Natural Resource Research, Bhutan (CoRRB) approved the survey and informed consent was obtained from all the participants. The study was conducted using three methodological approaches and adopted some of the items from consolidated criteria for Reporting of Qualitative research (COREQ) reporting guidelines for qualitative analysis [21].

1. Focus group discussion (expert group).
2. Collectors perception interview.
3. Population density survey.

### Focus group discussion (FGD)

This method was a preliminary step aimed at identification of priority medicinal plants by experts from different background. They were researchers from the Renewable Natural Resource Research and Development Centre, (RNRRDC), Yusipang, (a research institute mandated to also conduct study on high altitude medicinal plants), experts from Spices Medicinal and Aromatic Plants (SMAP) program (under Horticulture Division), Department of Agriculture (DoA) and traditional medicine experts and taxonomists from ITMS (Ministry of Health) and conservationists from Nature Conservation Division (NCD), Department of Forests and Park Services (DoFPS). These priority species were determined based on the concerns over the sustainability of harvesting and its long term capacity to meet the ever growing demand. The specific criteria to list top 16 species were;

1. Market value of the species.
2. Volume of species collected.
3. Number of collectors involved.
4. Abundance of the species in natural habitat.

### Collectors’ perception interview

The aim of this interview is to assess the level of awareness among the collectors with respect to value of the species, threat level, abundance, quantity collected. Participants were the residents of Lingshi who annually collect these medicinal plants. They were selected using purposive sampling method. Semi-structured questionnaire were used for face to face manner interview. A total of 19 key informants were interviewed in Dzongkha.^2^ None of the participants refused to participate and none other than interviewee were present at the time of interview. Free listing and preference ranking techniques were deployed to interview key informants (collectors) residing in Lingshi areas.

Data were collected on species the people collect the most, value (priority), areas, time of collection, status (threats and sustainability) and other uses if any (by the community). The questionnaires were pretested and accordingly changes were made prior to interview. The detail on collectors is shown below in Table 1.

**Table 1:** Characteristics of informant interviewed

Village	Collector	Age range	Sex
Chebesa	n = 2	f = 20, m = 52	f = 1, m = 1
Gangyul	n = 7	f (22–38), m (21–63)	m = 4, f = 3
Khakew	n = 1	m (20)	m = 1
Meseyul	n = 5	m (26–54)	m = 5
Shayul	n = 3	f (24,27,60)	f = 3
Zangthang	n = 1	m (38)	m = 1

‘N’, ‘m’ and ‘f’ refers to ‘number’, ‘male’ and ‘female’, respectively and their value within parenthesis

### Population density survey

The purpose of the population density survey was to cross validate the findings from focus group discussion and collectors perception interview. Two rounds of participatory surveys (along with collectors) were conducted to assess population density of the identified species in the field using quadrat. The quadrats were placed along the altitude gradients and each quadrat was divided into 100 sub-plots of 1 m^2^ and 10 × 10 m^2^ for herbs and shrubs, respectively. Data on ecological attributes (altitude range and slope gradient) were recorded. Given the very patchy and localised nature of the distribution of priority species, quadrat locations were selected in such a way to cover at least five plants of a selected priority species and to minimize subjective biasness, same person counted the plants in each quadrat (1 m^2^) throughout the survey. The place was marked to facilitate subsequent re-measuring using hand-held Garmin Etrex GPS units, and mapped.

In the case of plants that tend to occur in clumps, such as *Neopicrorhiza scrophulariiflora*, *Nardostachys grandiflora*, *Rhodiola crenulata*, *Chrysosplenium nudicaule* and *Gentiana urnula*, where it is not easy to judge what an individual plant is, both clump numbers and stem numbers were recorded. Herbarium specimens were sampled and brought to laboratory to ensure correct identification of species. Further, officials from the Jigme Dorji Wangchuck National Park joined the survey team to ensure compliance with the Nature Conservation Act of Bhutan, 1995.

## Data analyses

**Fig. 2:**
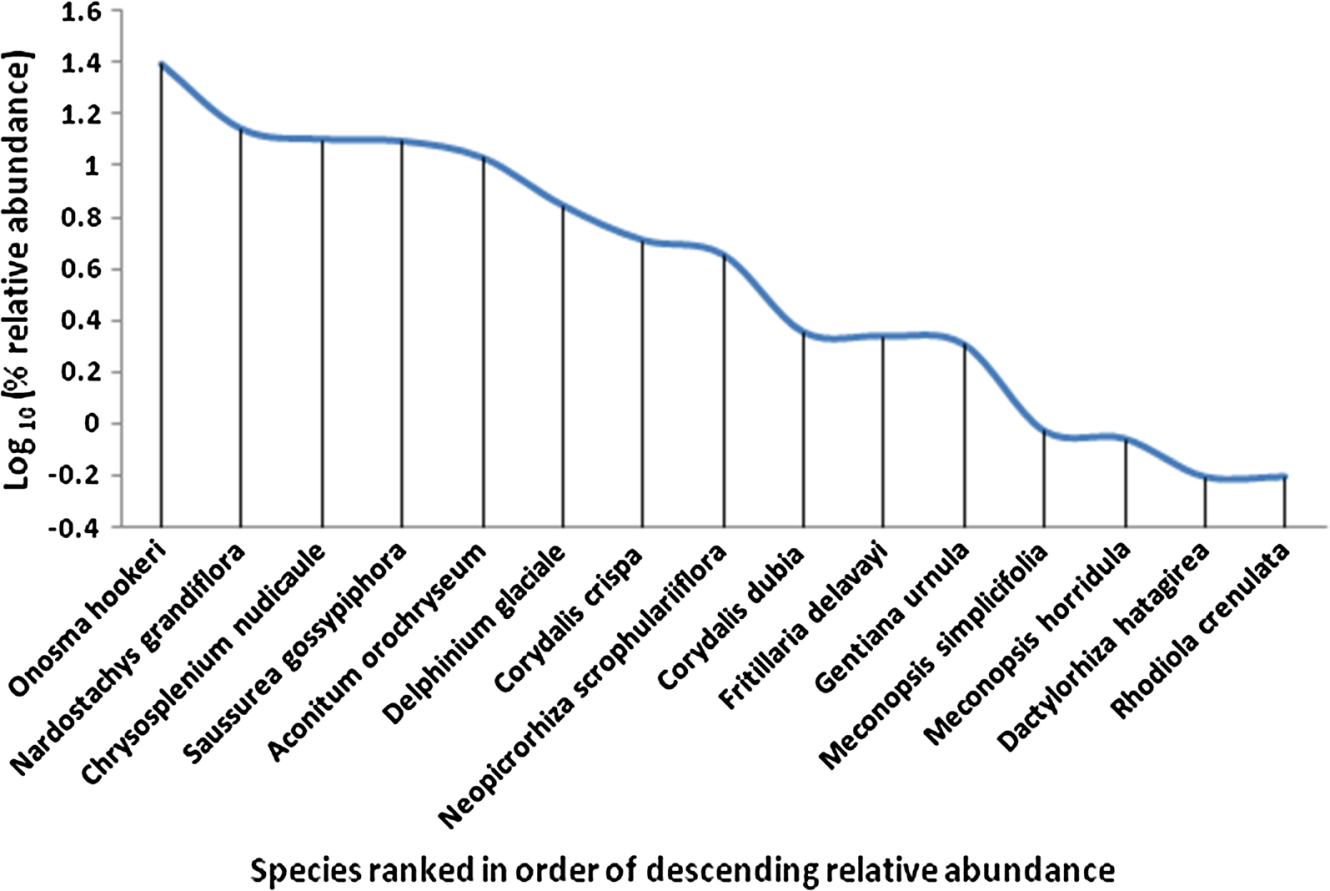
Whittakers plot for priority species

The information obtained from the expert group discussion was used in identification of priority species base on status (abundance, threat level, value and volume collected). The data from key informants’ perception interviews were manually compared and ranked (based on scores) using Microsoft spreadsheets. Similarly, data from plant population density survey (plant density, altitude, slope aspects) were used to describe the species’ ecological characteristics in natural area where indiscriminate and unscientific harvesting occurs. Further, the findings from these three methods (focus group discussion, collectors’ perceived status and the field surveys) were triangulated to validate and assess the collectors understanding on these species. The priority species population density is described using rank relative abundance—Whittaker plot (Fig. 2).

## Results

Medicinal plants species in high altitude areas serve as an important source of cash income for the local inhabitant. Due to high demand from Bhutan traditional medicine system (BTMS), there has been continuous harvesting in the wild for many years which has now threatened these medicinal plant species [22]. Expert group’s priority species and collector’s perception interview yielded similar results with 10 out of 16 priority species ranked as threatened. They collected species irrespective of its abundance, value and status. Survey on ecological status and species distribution showed highly variable plant density (0.04–15.9) plant m^−2^. Majority of the species perceived by collectors as threatened fall within 16 priority list identified by expert group except for *Halenia elliptica.* However, their priority rank for the status differed from plant density count. *Dactylorhiza hatagirea* which was as low as *Rhodiola crenulata* (0.4 plant m^−2^) did not feature in interview as a threatened status. *Halenia elliptica* and *Rhododendron anthopogon* were collected in large quantities by collectors and yet did not feature in the priority species list.

### Findings from expert group discussion

More than 53 species of medicinal plant species being used by the ITMS exist in Lingshi of which 16 were identified as the priority species based on market value of the species, volume of species collected, number of collectors involved and their abundance in natural habitat. This species were: *Aconitum orochryseum, Chrysosplenium nudicaule, Corydalis dubia, Corydalis crispa, Dactylorhiza hatagirea, Delphinium glaciale, Fritillaria delavayi, Gentiana urnula, Meconopsis horridula, Meconopsis simplicifolia, Nardostachys grandiflora, Onosma hookeri, Neopicrorhiza scrophulariiflora, Rhodiola crenulata, Saussurea gossypiphora, Veronica celiata*.

### Findings from collector’s perception interview

Of the 16 priority species, eight species (*Corydalis crispa*, *Nardostachys grandiflora*, *Rhodiola crenulata, Meconopsis simplicifolia, Neopicrorhiza scrophulariiflora, Onosma hookeri, Fritillaria delavayi and Meconopsis horridula* were found to be most abundant in Lingshi. However, the collectors collected mostly *Rhododendron anthopogon* (not in priority list) followed by *Corydalis crispa* (the most abundant species), *Meconopsis simplicifolia* (abundant, threatened, valuable), *Rhodiola crenulata* (abundant), *Delphinium glaciale* (threatened and valuable) and *Halenia elliptica* (not in priority list). Other most collected species included *Onosma hookeri* (threatened), *Chrysosplenium nudicaule* (threatened and valuable), *Neopicrorhiza scrophulariiflora* (abundant), *Nardostachys grandiflora (*abundant and valuable) and *Fritillaria delavayi* (abundant, threatened, and valuable). *Halenia elliptica* and *Rhododendron anthopogon* were neither included in priority list nor in any category (abundant, threatened and valuable).

Most collectors were aware that some medicinal plants are under threat. Species perceived to be the *most* threatened included *Onosma hookeri*, followed by *Corydalis dubia*, *Meconopsis simplicifolia*, *Gentiana urnula*, *Corydalis crispa*, *Delphinium glaciale* and *Halenia elliptica*. There were no predefined collecting areas for these priority species. Most of the collectors were nomads (yak herders) and collecting sites depended on where they herd their yaks. Thus, choice of the species they collected was influenced by the species’ availability within a reasonable walking distance from their grazing sites. No sense of permanent ownership is observed among collectors for a particular collection area.

Species dug up by the roots (*Corydalis dubia*, *Onosma hookeri*, *Gentiana sp.*, *Fritillaria delavayi* and *Nardostachys grandiflora*) appeared highly exploited. They also expressed concerns on the implications of collecting plants before seed set (referring to *Corydalis dubia*, *Meconopsis simplicifolia, Veronica celiata and Onosma hookeri* in this context), and an understanding that harvesting process for some species (e.g. *Rhodiola crenulata*) is wasteful as much of the material is discarded. *Dactylorhiza hatagirea* and *Gentiana urnula* were mentioned as species that are relatively rare and inaccessible.

Our assessment on the effect of yak (trampling and grazing) through perception revealed lack of significant conflict, although yaks were found to graze on a few species *(Rheum sp.* and *Codonopsis bhutanica*) that are not listed as priority species. Blue sheep were more often said to eat medicinal plants—particularly the higher-altitude species—but again this was not identified as a significant issue. Increased number of collectors was claimed by some informants to be a contributing factor to the decline of certain species (e.g. *Meconopsis horridula*, *Meconopsis simplicifolia*, *Delphinium glaciale*, *Onosma hookeri*, *Corydalis dubia* and *Corydalis crispa*). Table 2 shows collectors perception on ecological status and use of priority species.

**Table 2:** Detail ecological description of the priority species, their uses and status as perceived by the collectors

Species	Common name (Dzongkha)	Location description	Local name	Local use	Collectors’ perception score				Status
					Abundance	Threatened	Valuable	Most collected	
*Aconitum orochryseum*	Bongkar	Found in heavily grazed grasslands; Churki, Sepchu, Walethang and Geshoy	Unknown	Unknown	0	5	0	0	Rare and threatened
*Chrysosplenium nudicaule*	Yakima	Grassland and occasionally Rhodendron; Chewla, Yakla and Chewphu	Unknown	Unknown	13	1	0	5	Most abundant and 2nd most collected
*Corydalis dubia*	Rezeun	Grassland of Chebesa, Gangyul and Lingshi Dzong	Reken	Unknown	4	4	4	1	Abundant, threatened, valuable but rarely collected
*Corydalis crispa*	Bashaka	Fine and shallow gravels; Chebesa, Gangyul	Unknown	Eaten by yak	11	8	7	5	Most abundant, also threatened, valuable, and most collected
*Dactylorhiza hatagirea*	Wangla	Found in grassland (often with scattered *Rhododendron*, *Salix* and/or *Juniperus*) and dense herbaceous vegetation Lingshi, Naro	Ja ola Omla	Rhizome eaten as food	0	4	9	0	Rare, threatened, 3rd most valuable, and rarely collected
*Delphinium glaciale*	Jagodpoe	Scree slopes Boeza, Chewla and Lolung (Chewphu)		Use as incense and also eaten by Blue sheep	5	8	12	1	Abundant
*Fritillaria delavayi*	Tseka	Scree slopes of Chebesa and Gangyul		Consumed as vegetable, seed and bulb also eaten, flowers grazed by blue sheep	0	8	7	0	Rare, threatened and hardly collected
*Gentiana urnula*	Gangachung	Steep scree slopes (small to medium size stones) Chebesa and Gangyul		Eaten by yak	11	0	1	2	Most abundant,
*Meconopsis horridula*	Tserngon	Rocky slopes with a mixture of scree and patches of grasses, herbs and mosses	Omrim		18	7	2	8	Most abundant, threatened, medium value and most collected
*Meconopsis simplicifolia*	Udpel sngonpo	Relatively widespread in slopes and in valley usually on damp ground in small but only in scattered patches	Geche metog		0	4	5	4	Rare, threatened, medium value and occasionally collected
*Nardostachys grandiflora*	Pangpoe	Open grassland and sparsely among Rhododendron shrubs		Use as incense	0	9	13	0	Rare, threatened, high value, rarely collected
*Onosma hookeri*	Dimug	Grassland area of scattered *Juniperus*	Muksi	Use as religious dye for ceremony	–	–	1	–	–
*Neopicrorhiza scrophulariflora*	Putishing	Relatively widespread among Thododendron and small wood clearing		Use as decoction to cold	–	–	1	–	–
*Rhodiola crenulata*	Solo marpo	Yangtay, Chewla, Yakla, Boeza and Chewphu on steep, rocky, scree slopes or boulder slopes with scattered patched of herbaceous vegetation and mosses	Tsemarp, lamichop		–	8	11	2	Rare, threatened, high value and occasionally collected
*Saussurea gossypiphora*	Jagod supa	Found scattered on patches of grassland and slopes with scree and moss		Eaten by blue sheep and also for filling pillows in past	17	2	5	2	Most abundant, low value and rarely collected
*Veronica celiata*	Domnag domthri	On grazed grassland on gentle slopes			7	12	9	3	Scarce, most threatened, medium value and occasionally collected

### Findings from field survey

**Fig. 3:**
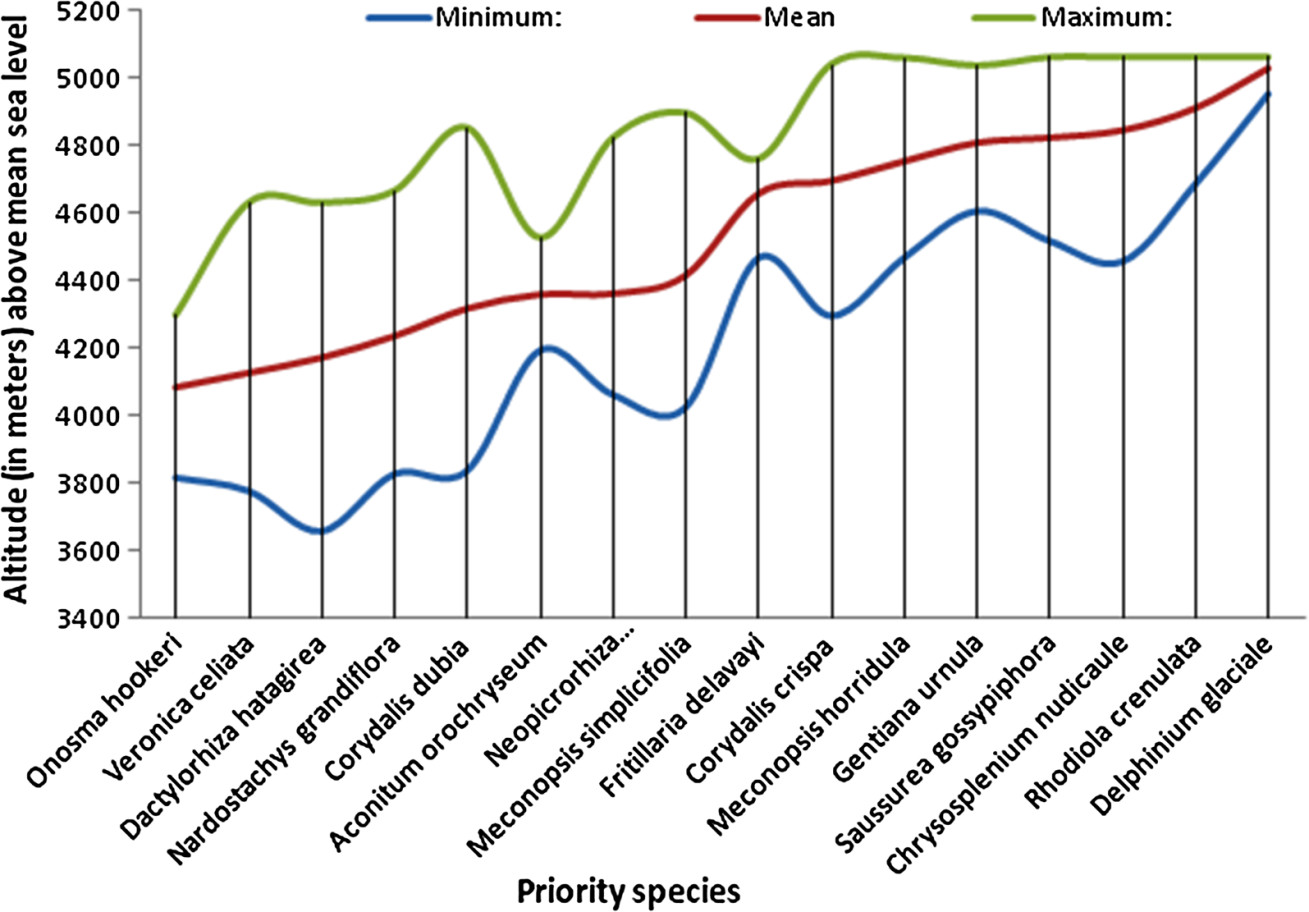
Distribution of the priority species base on altitude

Among the 16 priority species, *Onosma hookeri* (15.9 plants m^−2^) was the most abundant species followed by *Nardostachys grandiflora*. *Chrysosplenium nudicaule* and *Saussurea gossypiphora* rank third in terms of rank abundance but also revealed high evenness (8–8.9 plants m^−2^). Similarly, high evenness in distribution was also observed in these three species (*Corydalis dubia, Fritillaria delavayi, Gentiana urnula*) but they were relatively scarce in distribution with (1.3–1.5) plants m^−2^. Low evenness among species existed between *Onosma hookeri* (15.9 plants m^−2^) and *Nardostachys grandiflora* (8.9 plants m^−2^), *Neopicrorhiza scrophulariiflora* (and *Corydalis dubia* and between level of *Gentiana urnula* and *Meconopsis simplicifolia.* On the other hand, *Rhodiola crenulata* and *Dactylorhiza hatagirea* (0.4 plants m^−2^) are the rarest among the priority species, followed by *Meconopsis horridula* and *Meconopsis simplicifolia*. *Corydalis dubia, Fritillaria delavayi, Gentiana urnula* (1.4 plants m^−2^) are some of the species in second category rarer species among the priority species identified. Slope aspects, associated species and vegetation differed from species to species. The effect of altitude on the distribution is shown in Fig. 3.

Summary of the survey on each species is presented Table 3 and comparison of collectors’ perception on ecological status with plant density for 16 priority species is shown in Table 4.

**Table 3:** Ecology and status of priority species as obtained from survey

Species	Location	Plants per m^−2^	Altitude range (m)	Aspects	Associated species	Vegetation	Status
*Dactylorhiza hatagirea*	Lingshi, Naro	0.4	3654–4629	N- to NE	*Bistorta macrophylla*, *Anemone rivularis* and *Nardostachys grandiflora* in the Lingshi area, and with *Aconitum orochryseum* in Naro	Found in grassland (often with scattered *Rhododendron*, *Salix* and/or *Juniperus*) and dense herbaceous vegetation	Concerning
*Rhodiola crenulata*	Yangtay, Chewla, Yakla, Boeza and Chewphu	0.4	4685–5060	SE- [through S] to NW- facing	*Delphinium glaciale*, *Meconopsis horridula*, *Saussurea goaggypiphora*, *Chrysosplenium nudicaule*, *Eriophyton wallichii* and *Pleurospermum amabile*	Found in grassland and other herbaceous vegetation on high-altitude boulder slopes with scree and moss	Occurred in very few sites
*Meconopsis horridula*	Rocky slopes	0.56	4466–5058	SW- to NW-facing	*Chrysosplenium nudicaule*, *Rhodiola crenulata*, *Saussurea gossypiphora* and *Eriophyton wallichii*	Found in scree and patches of grasses, herbs and mosses	
*Meconopsis simplicifolia*	Slopes and in valley b	0.6	4022–4895		*Rubus* sp. and *Rhodiola* sp	Found in shrubby vegetation and woodland	Patchy with high demand and scarce
*Gentiana urnula*		1.3	4605–5035	NW-facing	*Eriophyton wallichii*, and was also found with *Gentiana urnula*, *Cremanthodium reniforme* and *Meconopsis horridula*	Found in steep scree slopes (small-to medium-sized stones—mainly reddish sandstone)	
*Fritillaria delavayi*		1.4	4466–4759	SW- to W-facing	*Eriophyton wallichii* and often with *Corydalis dubia* and *Gentiana urnula*	Found in scree slopes	Scarce and difficult to collect
*Corydalis dubia*		1.45	4294–5039	SW- to NW-facing	*Eriophyton wallichii*, *Cremanthodium reniforme*, *Fritillaria delavayi* and *Saussurea gossypiphora*	Found in Grassland	Under threat
*Neopicrorhiza scrophulariflora*	Relatively widespread	2.9	4060–4821	N- to NE	*Rhododendron anthopogon*, *Bistorta macrophylla* and *Thalictrum reniforme*	Found in *Rhododendron* scrub and in small woodland clearings, often on mossy ground	
*Corydalis crispa*		3.3	3834–4851		*Polygonum* sp. *Eriophyton wallichii*, *Tanacetum nubigenum*, *Erysimum bhutanicum* and *Phlomis rotata*	Found in fine and shallow gravels	
*Delphinium glaciale*	Boeza, Chewla and Lolung (Chewphu)	4.5	4953–5060	Commonly SE- to SW- but also NW-facing	*Rhodiola crenulata*, and also occurs with *Eriophyton wallichii*, *Gentiana urnula*, *Stellaria decumbens* and *Saussurea* spp	Found in scree slopes	Depleted
*Veronica celiata*	Grazed grassland	5.3	3774–4629	SW- to SE-facing (or NE-facing)	*Pedicularis oliverina*, *Anemone rivularis*, *Geranium pratense* and *Thermopsis barbata*	Found in grazed grassland, sometimes with scattered *Rhododendron* and *Juniperus*	Occurs restricted, difficult although high value harvest seed sets
*Aconitum orochryseum*	Churki, Sepchu, Walethang and Geshoy	6.9	4191–4526	SW-NW	*Geranium refractum*, *Bistorta macrophylla*, *Pedicularis siphonantha*	Found in heavily grazed grasslands	
*Saussurea gossypiphora*	Scattered patches	8	4516–5060	Not specific	*Chrysosplenium nudicaule*, *Meconopsis horridula*, *Rhodiola crenulata*, *Bistorta macrophylla*, *Corydalis dubia* and *Eriophyton wallichii*	Found in grassland and other herbaceous vegetation on high-altitude boulder slopes with scree and moss	Collected by very few people
*Chrysosplenium nudicaule*	Chewla, Yakla and Chewphu	8.13	4455–5060	Steep slope (NW to SW)	*Meconopsis horridula*, *Saussurea gossypiphora* and *Rhodiola crenulata*	Found in grassland and occasionally Rhodendron	
*Nardostachys grandiflora*	Open grassland	8.9	3824–4664	NE-facing lesser degree E- and SE-facing	*Bistorta macrophylla*, *Dactylorhiza hatagirea*, *Morina nepalensis*, *Onosma hookeri*	Found in open grassland but also among sparse *Rhododendron* scrub	
*Onosma hookeri*	Grassland with scattered *Juniperus*	15.9	3813–4296	SW- to SE-facing	*Pterocephalus hookeri*, *Bistorta macrophylla*, *Anemone rivularis* and *Nardostachys grandiflora*	Found in grassland with scattered *Juniperus*	Patchy and confined to narrow altitude range

**Table 4:** Comparison of collectors’ perception on ecological status with plant density for 16 priority species

Species	Survey			Collectors’ perception score				
	Plants per m^−2^	Altitude range (masl)	Observed status	Abundance	Threatened	Valuable	Most collected	Status
*Dactylorhiza hatagirea*	0.40	3654–4629	Concerning	0	5	0	0	Rare and threatened
*Rhodiola crenulata*	0.40	4685–5060	Occurred in very few sites	13	1	0	5	Most abundant and 2nd most collected
*Meconopsis horridula*	0.56	4466–5058	–	4	4	4	1	Abundant, threatened, valuable but rarely collected
*Meconopsis simplicifolia*	0.60	4022–4895	Patchy distribution with high demand and scarce	11	8	7	5	Most abundant, also threatened, valuable, and most collected
*Gentiana urnula*	1.30	4605–5035	–	0	4	9	0	Rare, threatened, 3rd most valuable, and rarely collected
*Fritillaria delavayi*	1.40	4466–4759	Scarce and difficult to collect	5	8	12	1	Abundant
*Corydalis dubia*	1.45	4294–5039	Under threat	0	8	7	0	Rare, threatened and hardly collected
*Neopicrorhiza scrophulariflora*	2.90	4060–4821	–	11	0	1	2	Most abundant,
*Corydalis crispa*	3.30	3834–4851	–	18	7	2	8	Most abundant, threatened, medium value and most collected
*Delphinium glaciale*	4.50	4953–5060	Depleted	0	4	5	4	Rare, threatened, medium value and occasionally collected
*Veronica celiata*	5.30	3774–4629	Occurs restricted, difficult although high value harvest seed sets	0	9	13	0	Rare, threatened, high value, rarely collected
*Aconitum orochryseum*	6.90	4191–4526	–	–	–	1	–	–
*Saussurea gossypiphora*	8.00	4516–5060	Collected by very few people	–	–	1	–	–
*Chrysosplenium nudicaule*	8.13	4455–5060	–	–	8	11	2	Rare, threatened, high value and occasionally collected
*Nardostachys grandiflora*	8.90	3824–4664	–	17	2	5	2	Most abundant, low value and rarely collected
*Onosma hookeri*	15.90	3813–4296	Patchy and confined to narrow altitude range	7	12	9	3	Scarce, most threatened, medium value and occasionally collected

The figure under “Collectors’ perception score” refers to score provided by collectors with respect to its category (abundant, threatened, valuable and most collected)

## Discussion

The main purpose of the expert group discussion was to identify species that are over exploited and need immediate attention from the stakeholders. The discussion with expert group gave direct indication for our basis on identifying of 16 priority species on which to study. Although, majority of the herbs used in ITMS are collected from Lingshi, only 53 species were reflected during the discussion. Collectors perceptions tally to an extent that most of the priority species identified by expert group are at risks due to over exploitation. This is because they were either collected by a large number of collectors (in large quantity) without any management or harvesting plan.

Most medicinal plants were collected between the months of June and August. Some of the plants are harvested before seed set, directly impacting the multiplication rate while whole plants are uprooted for few some species. Several collectors made a direct link between the apparent decline of these plants and the level and manner of their collection. Key issues identified by the collectors were the non-sustainability of current collecting methods and the difficulties in managing a common resource in a controlled and sustainable manner.

Collectors were found to obtain permit for those abundant species that coincides with their yak grazing schedule. Nevertheless, allotted permits for several species remain distributed among a number of collectors, resulting in more than one person harvesting the same plant species population independently. Consequently, any sustainability measures that may be followed by an individual (e.g. collecting up to a certain quantity or restricting to a certain proportion of a population), are likely to be negated by the collectors those follow behind.

BTM is the primary market for medicinal plants that can influence the sustainability of collection in the region, thus helping to ensure the future of important medicinal plant populations. However, if ITMS only commands a limited proportion of the market, then its potential for leverage is much lower. It is very likely that collection for ITMS is only a part of the picture. It is, however, difficult to determine whether or not this is the case, and even harder to quantify it. In the interviews, all collectors claimed that they only gathered medicinal plants for ITMS, but given that any other plant collection would technically (under current regulations) be illegal, this is not surprising.

Although, most of the collectors were aware on the scientific method of harvesting, implementation of sustainable harvesting approach has been a complex issue (involving ecological, biological, social and economical factors) [12]. Certain species are much more restricted in their distribution reflecting ecological requirements, which may be very specific. Considering altitude, for example, the species with the most restricted range (*Delphinium glaciale*) is likewise the most geographically restricted (occurring at three sites only). This species also shows the most limited range of slope angle at the survey sites. Conversely *Corydalis crispa*, *Nardostachys grandiflora*, *Dactylorhiza hatagirea* and *Meconopsis simplicifolia* all show broad ranges of altitude and slope conditions and are relatively widely distributed across the region. *Nardostachys grandiflora* DC., *Neopicrorhiza scrophulariiflora* (Pennell) D.Y. Hong*, Onosma hookeri* Clarke and *Fritillaria delavayi* Franchet [23] whose roots and rhizomes are collected from Lingshi for traditional medicine production, attains rapid recovery through vegetative propagation [20].

Most of the priority species surveyed showed a distinctly patchy distribution. Even the relatively abundant and widespread species tend to occur in small, restricted populations, making any sort of large-scale estimate of population impossible. Thus, population density data presented cannot realistically be used to provide an overall estimate of the regional resource for each species owing to very patchy distribution and our sampling method (quadrats with at least five species listed by expert group were considered). Therefore, actual species density in the field is much lower than presented here. However, our findings do provide an important insight into species status in the main collecting areas, and baselines for future monitoring.

## Conclusions

Medicinal plants and their associated traditional healing systems have important roles as national heritage and due recognition and their visibility in national policies are imperative. As long as such healing system exists, wild harvest of plants will continue. Thus, long-term sustainability of medicinal plant resources in the wild will have profound implications.

Ranking the priority species through collector’s interviews and expert group (free listing) yielded similar results. Both the methods (expert group discussion and interviews) ranked *Corydalis crispa, Nardostachys grandiflora, Rhodiola crenulata, Meconopsis simplicifolia, Neopicrorhiza scrophulariiflora and Onosma hookeri* as most abundant species. Most of the collectors are aware of the status and implications of collecting of these priority species. Some species under threat (*most* threatened) included—*Onosma hookeri*, *Corydalis dubia*, *Meconopsis simplicifolia*, *Gentiana urnula*, *Corydalis crispa*, *Delphinium glaciale* and *Halenia elliptica*. Species dug up by the roots (*Corydalis dubia*, *Onosma hookeri*, *Gentiana sp.*, *Fritillaria delavayi* and *Nardostachys grandiflora*) expressed to be highly exploited. *Dactylorhiza hatagirea* and *Gentiana urnula* are two species that became relatively rare and inaccessible.

Survey results and collectors’ perceptions tally to an extent with respect to status and ecology. Survey results also show that species such as *Dactylorhiza hatagirea, Rhodiola crenulata, Meconopsis simplicifolia and Meconopsis horridula*, were critically low in plant density with less than a plant per m^2^ while *Delphinium glaciale Fritillaria delavayi and Aconitum orochryseum* are confined to narrow altitude range. Implementation of effective management plans on sustainable harvest prepared in collaboration and with communities and capacity building on the reproductive biology of these priority species were found essential. Numerous parameters affect wild harvest of these species. A wider understanding amongst stakeholders (legislative and governmental bodies, including non-governmental organizations (NGOs), ITMS, SMAP and community) on the ecological status and significance will help bring forth better results in sustaining wild harvest. Domestication and cultivation of these high altitude species is still a major challenge both in terms of replicating high altitude growing environment and economic feasibility besides the beliefs.

## Additional files

10.1186/s12898-016-0100-1 Geo-location, vegetation cover and habitat description for priority species at different survey points.

10.1186/s12898-016-0100-1 Priority species plant density at different survey points.

## Abbreviations

BTM: Bhutan traditional medicine
CoRRB: Council for Renewable Natural Resource Research, Bhutan
DoA: Department of Agriculture
DoFPS: Department of Forests and Park Services
FGD: focus group discussion
GNH: gross national happiness
ITMS: Institute of traditional medicine services
IUCN: International union for conservation of nature and natural resources
Masl: meter above sea level
NCD: Nature Conservation Division
RNRRDC: Renewable Natural Resource Research and Development Center
RGoB: Royal Government of Bhutan
SMAP: Spices Medicinal and Aromatic Plants
TM: Traditional medicine
WHO: World Health Organization
WWF: World Wildlife Fund

## Footnotes

1 Local administrative unit (under district).

2 An official and the national language of Bhutan.

## References

[1] Iqbal M: International trade in non-wood forest products: an overview. 1993. http://www.fao.org/docrep/x5326e/x5326e00.htm. Assessed 23 May 2014.

[2] Schippmann U, Leaman D, Cunningham AB. A comparison of cultivation and wild collection of medicinal and aromatic plants under sustainability aspects. In: Medicinal and aromatic plants: agricultural, commercial, ecological, legal, pharmacological and social aspects. 2006. http://library.wur.nl/ojs/index.php/frontis/article/view/1225. Assessed 15 Mar 2014.

[3] Hamilton AC, Radford EA. Identification and conservation of Important Plant Areas for medicinal plants in the Himalaya. Plantlife International (Salisbury, UK) and Ethnobotanical Society of Nepal(Kathmandu, Nepal). 2007

[4] Larson HO, Olsen CS (2007). Unsustainable collection and unfair trade? Uncovering and assessing assumption regarding central Himalayan medicinal plant conservation. Biodiver Conserv..

[5] Committee QCRS: Flora collection. In: Queensland CRA/RFA Steering Committee; 1998. http://www.daff.gov.au/SiteCollectionDocuments/rfa/regions/qld-south-east/resources/qld_se_raa_se4.2flor.pdf. Assessed 15July 2014.

[6] Rasul G, Choudhary D, Pandit BH, Kollmair M (2012). Poverty and livelihood impacts of a medicinal and aromatic plants project in India and Nepal: an assessment. Mt Res Dev.

[7] Wangchuk P, Tobgay T (2015). Contributions of medicinal plants to the gross national happiness and biodiscovery in Bhutan. J Ethnobiol Ethnomed.

[8] Wangchuk D. An introduction to traditional medicine services in Bhutan, Institute of Traditional Medicine Services; 2010. http://www.nitm.edu.bt/images/Publications/TMR/AN%20INTRODUCTION%20TO%20TRADITIONAL%20MEDICINE%20SERVICES%20IN%20Btn3rdEdtn.pdf. Assessed 21 Feb 2014.

[9] Wangchuk P, Pyne SG, Keller PA (2011). Ethnobotanical authentication and identification of Khrog-sman (lower elevation medicinal plants) of Bhutan. J Ethnopharmacol.

[10] Wangchuk P, Wangchuk D, Aagaard-Hansen J. Traditional Bhutanese medicine (gSo-BA Rig-PA): an integrated part of the formal health care services. 2007. http://researchonline.jcu.edu.au/32785/. Assessed 12 Mar 2015. 17539263

[11] Lhamo N, Nebel S (2011). Perceptions and attitudes of bhutanese people on Sowa Rigpa, traditional bhutanese medicine: a preliminary study from Thimphu. J Ethnobiol Ethnomed..

[12] Ugyen P, Olsen A. Vulnerable medicinal plants and the risk factors for their sustainable use in Bhutan. 2009. http://www.bhutanstudies.org.bt/publicationFiles/JBS/JBS_Vol19/19-6.pdf. Assessed 18 Feb 2015.

[13] Rai L, Prasad P, Sharma E (2000). Conservation threats to some important medicinal plants of the Sikkim Himalaya. Biol Conserv.

[14] Chauhana S, Nautiyala B, MC Nautiyala R. Trade of threatened Himalayan medicinal and aromatic plants-socioeconomy, management and conservation issues in Garhwal Himalaya, India. 2013. https://globaljournals.org/GJMR_Volume13/2-Trade-of-Threatened-Himalayan.pdf. Assessed 12 Aug 2014.

[15] Kala CP (2005). Indigenous uses population density, and conservation of threatened medicinal plants in protected areas of the Indian Himalayas. Conserv Biol..

[16] Kumar GP, Kumar R, Chaurasia O, Singh SB (2011). Current status and potential prospects of medicinal plant sector in trans-Himalayan Ladakh. J Med Plants Res..

[17] Wangchuk P (2009). High altitude medicinal plants of Bhutan: an illustrated guide for practical use: Pharmaceutical and Research Unit.

[18] Barakoti TP, Plaza S. Country Status Report on Medicinal and Aromatic Plants in Nepal. In: Expert Consultation on Promotion of Medicinal and Aromatic Plants in the Asia-Pacific Region: Proceedings: 2013. http://www.egfar.org/sites/default/files/medicinal_and_aromatic_plants-proceedings.pdf. Assessed 19 Apr 2015.

[19] Singh H, Husain T, Agnihotri P, Pande P, Khatoon S (2014). An ethnobotanical study of medicinal plants used in sacred groves of Kumaon Himalaya, Uttarakhand. India. J Ethnopharmacol..

[20] Ticktin T (2004). The ecological implications of harvesting non-timber forest products. J Appl Ecol.

[21] Tong A, Sainsbury P, Craig J (2007). Consolidated criteria for reporting qualitative research (COREQ): a 32-item checklist for interviews and focus groups. Int J Qual Health Care.

[22] Expert consultation on Promotion of medicinal and aromatic Plants in the Asia-Pacific Region. http://www.apaari.org/wp-content/uploads/downloads/2014/10/Medicinal-and-Aromatic-Plants-Proceedings_21-10-2014-1.pdf. Accessed 12 Jan 2016.

[23] Airi S, Rawal RS, Dhar U, Purohit AN (2000). Assessment of availability and habitat preference of Jatamansi—a critically endangered medicinal plant of west Himalaya. Curr Sci.

[24] Forest and Nature Conservation Act of Bhutan, 1995, http://www.bafra.gov.bt/wp-content/uploads/2015/06/ForestNatureConAct1995.pdfAccessed 1 Jan 2012.

[25] WHO, IUCN & WWF: Guidelines on the conservation of medicinal plants. http://ruralnetwork.ca/sites/default/files/tools_resources/who-1988-medicinal-plant-guidelines.pdf. Accessed 17 Aug 2012.

